# Pumilio2 Promotes Growth of Mature Neurons

**DOI:** 10.3390/ijms22168998

**Published:** 2021-08-20

**Authors:** Rico Schieweck, Elisa-Charlott Schöneweiss, Max Harner, Daniela Rieger, Christin Illig, Barbara Saccà, Bastian Popper, Michael A. Kiebler

**Affiliations:** 1 Biomedical Center (BMC), Department for Cell Biology & Anatomy, Medical Faculty, Ludwig-Maximilians-University, 82152 München, Germany; Rico.Schieweck@med.uni-muenchen.de (R.S.); max.harner@med.uni-muenchen.de (M.H.); daniela.rieger@bmc.med.lmu.de (D.R.); Ch.Illig@web.de (C.I.); michael.kiebler@med.uni-muenchen.de (M.A.K.); 2 Zentrum für Medizinische Biotechnologie (ZMB), University of Duisburg-Essen, 41541 Duisburg, Germany; elisa-charlott.schoeneweiss@fujifilm.com (E.-C.S.); barbara.sacca@uni-due.de (B.S.); 3 Biomedical Center (BMC), Core Facility Animal Models, Ludwig-Maximilians-University, 82152 München, Germany

**Keywords:** Pumilio2, neuronal growth, eIF4E, atomic force microscopy

## Abstract

RNA-binding proteins (RBPs) are essential regulators controlling both the cellular transcriptome and translatome. These processes enable cellular plasticity, an important prerequisite for growth. Cellular growth is a complex, tightly controlled process. Using cancer cells as model, we looked for RBPs displaying strong expression in published transcriptome datasets. Interestingly, we found the Pumilio (Pum) protein family to be highly expressed in all these cells. Moreover, we observed that Pum2 is regulated by basic fibroblast growth factor (bFGF). bFGF selectively enhances protein levels of Pum2 and the eukaryotic initiation factor 4E (eIF4E). Exploiting atomic force microscopy and in vitro pulldown assays, we show that Pum2 selects for *eIF4E* mRNA binding. Loss of Pum2 reduces eIF4E translation. Accordingly, depletion of Pum2 led to decreased soma size and dendritic branching of mature neurons, which was accompanied by a reduction in essential growth factors. In conclusion, we identify Pum2 as an important growth factor for mature neurons. Consequently, it is tempting to speculate that Pum2 may promote cancer growth.

## 1. Introduction

Cells, in particular neurons, control their proteome through a network of different RNA-binding proteins (RBPs) [1]. To date, several hundred RBPs have been identified [2,3,4]. Thus, it is not surprising that RBPs control many—if not all—pathways to maintain physiological homeostasis [1]. RBPs have been involved in many cellular processes, e.g., regulating splicing, RNA export/transport, decay, and translation [1]. In this context, the translation regulator Pumilio2 (Pum2) is of particular interest [5]. Pum2 regulates neurogenesis, neuronal differentiation [6,7,8], and brain homeostasis [9,10]. In particular, it controls axon outgrowth [8], dendritic branching, and synapse formation [11]. Therefore, Pum2 has an impact on a broad range of developmental and mature stages as well as cellular functions. Importantly, all these stages rely on the cell’s ability to regulate growth in distinct contexts. For example, it has been reported that Pum1 and Pum2 regulate body size [12]. In line with these observations are studies in cancer cells. Here, Pum2 regulates the oncogene *E2F3* [13]. Moreover, it is important for glioblastoma cell proliferation and stemness maintenance of breast cancer cells [14,15]. These findings strongly suggest that Pum2 has an impact on cellular growth, not only in neurons. Importantly, translation is a major determinant of growth [16,17]. Interestingly, Pum2 has a broad impact on protein synthesis in cortical neurons [18]. In line with this observation is the finding that Pum2 competes with the eukaryotic initiation factor 4E (eIF4E) for mRNA cap binding in *Xenopus* oocytes [19]. Supportively, it has been shown that Pum2 interacts with the translation repressor eukaryotic initiation factor 4E transporter (Eif4enif1 or 4E-T) to regulate neuronal differentiation [7]. Moreover, it interacts with the translation repressor fragile-X mental retardation protein (FMRP) [6]. Thus, it is plausible that Pum2 regulates growth through translation control of its targets. The link between translation regulators and growth is particularly interesting as nerve cells remodel their local nascent proteome in response to different cues including Netrin-1, Brain-derived neurotrophic factor (BDNF), and Semaphorin 3A (Sema3A) [20].

In the current study, we tested the interesting working hypothesis that Pum2 regulates neuronal growth. We found that Pum2, but not Staufen2 (Stau2), another well-studied RBP, responds to basic fibroblast growth factor (bFGF) but not to epidermal growth factor (EGF) or neuronal growth factor (NGF) treatment in cultured cortical neurons. Interestingly, bFGF increases levels of Pum2 and eIF4E, an important regulator of proliferation and growth [17,21]. Thereby, Pum2 activates *eIF4E* translation. Accordingly, we found that Pum2 knock-down (KD) decreases cell body size and dendritic branching of mature neurons. Complementary, and in line with published data [11,22], we show that Pum2 selects for *eIF4E* mRNA binding using atomic force microscopy. Together, our data suggest that Pum2 is a crucial regulator of neuronal growth. Moreover, it is tempting to speculate that it also contributes to growth and proliferation of cancer cells identifying Pum2 as novel oncotarget for therapies.

## 2. Results

### 2.1. bFGF Selectively Regulates Pum2 Protein Levels

**Figure 1:**
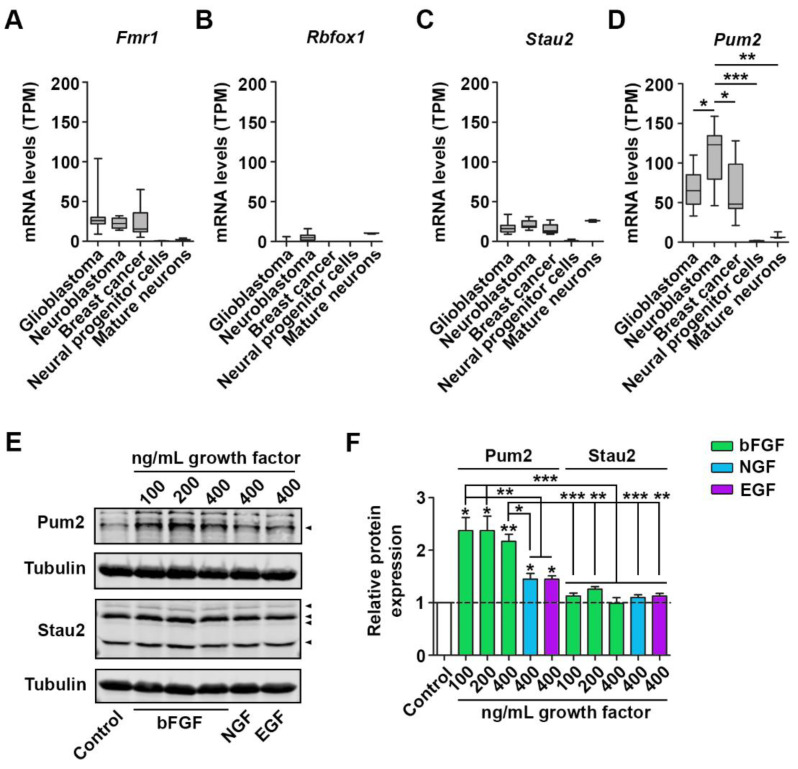
Neuronal Pum2 expression is stimulated by bFGF. (**A**–**D**) mRNA levels as transcripts per million (TPM) of FMRP (*Fmr1*) (**A**), *Rbfox1* (**B**), *Stau2* (**C**), and *Pum2* (**D**) in different cancer cells as well as in healthy neural progenitor cells and mature neurons. (**E**,**F**) Representative immunoblots Pum2 and Stau2 € with respective quantifications (**F**) upon incubation of mature (14 DIV) cortical neurons with bFGF, NGF, and EGF, respectively, for two days. β-III-Tubulin was used as loading control. Arrow heads indicate the respective proteins. *p*-values were calculated using Kruskal–Wallis test with Dunn’s Multiple Comparison Test (**D**), one-sample t-test within samples and Tukey’s Multiple Comparison Test between samples (**F**). * *p* < 0.05, ** *p* < 0.01, *** *p* < 0.001, 4 biological replicates.

To identify RBPs that might regulate cell growth, we reanalyzed published RNA-seq data from different breast cancer, glioblastoma, and neuroblastoma cell lines [23] as these cells display high growth and proliferation rates. Thereby, we focused on RNA expression of key RBPs. Among other RBPs such as Elav-like (Elavl) proteins, Staufen1, and RNA binding protein fox-1 homolog 2 (Rbfox2), we found the Pumilio protein family consisting of Pum1 and Pum2 to be highly expressed in glioblastoma, neuroblastoma, and breast cancer cells (Figure S1A–C). Moreover, we compared cancer cell expression with mRNA levels in healthy neural progenitor cells [24] and mature neurons [18] for essential neuronal RBPs such as FMRP, RNA-binding protein fox-1 (Rbfox1), Staufen2 (Stau2), and Pum2 (Figure 1A–D). Among these RBPs, *Pum2* mRNA exhibited selective and significant enrichment in cancer cells compared to neural progenitor cells or mature neurons. In contrast, Rbfox1, FMRP, and Stau2 were either not enriched in cancer cells or showed reduced expression compared to Pum2 (Figure 1D). Due to the high expression of *Pum2* in cancer cells and their great potential to growth and division, we speculated that Pum2 regulates growth in cells.

From these analyses, we concluded that Pum2 might enhance cellular growth. As it has important functions in nerve cells [8,10,11], we next asked whether Pum2 is also involved in regulating neuronal growth. Unlike cancer cells, neurons rely on extrinsic signals that regulate their growth and guide their differentiation. Consequently, nerve cells are very sensitive to growth factors [20]. Therefore, we first tested whether neuronal Pum2 respond to different growth factors. For this purpose, we incubated cortical neurons with bFGF and analyzed its effect on protein expression of essential neuronal RBPs such as Pum2 and Stau2 (Figure 1E,F). Interestingly, we found that bFGF treatment induces strong upregulation of Pum2 (Figure 1E,F). To control whether the effect of bFGF is specific or a general hallmark of growth factors, we also tested used NGF and EGF. In contrast to bFGF, neither NGF nor EGF induced a similar increase in Pum2 levels. Moreover, the bFGF effect was specific for Pum2 as Stau2 remained unaffected (Figure 1E,F). Similar to Pum2, we observed a strong upregulation of Rps6 phosphorylation in response to bFGF but not NGF or EGF treatment (Figure S1D,E), indicating that the mTOR pathways is also stimulated under these conditions. We also observed a tendency towards higher Rps6 protein levels upon bFGF incubation (Figure S1F,G). This trend, however, did not reach statistical significance. Together, our results point towards a role of Pum2 in regulating neuronal growth. Supportive for this notion are recent findings showing that Pum2 regulates axonal outgrowth [8], dendrite formation [11], and body size [12].

### 2.2. Pum2 Promotes Growth of Mature Neurons

**Figure 2:**
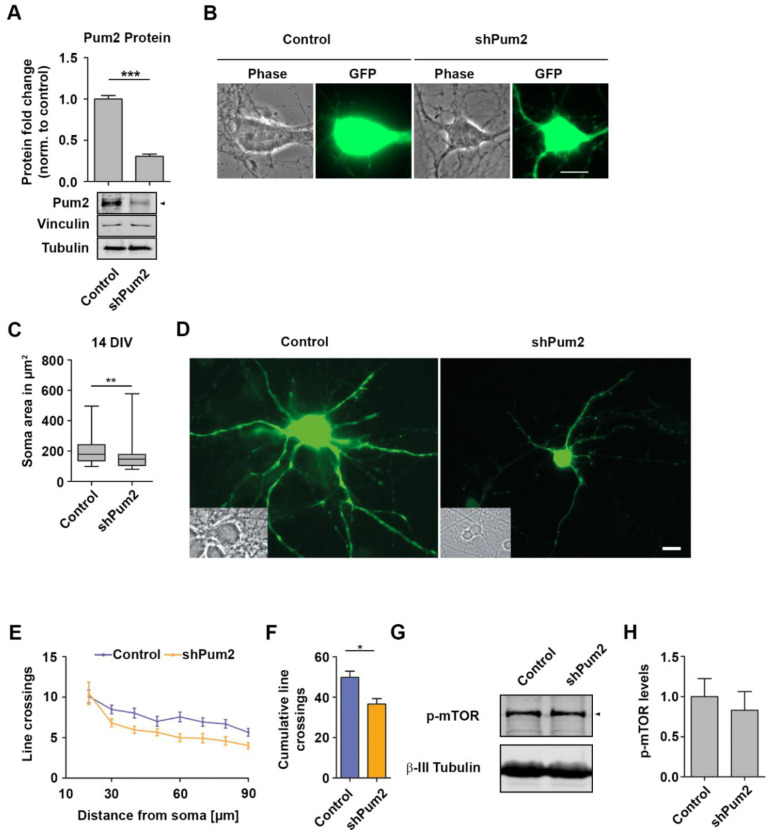
Pum2 promotes growth of mature neurons. (**A**) Representative immunoblot against Pum2 from shControl and shPum2 transduced cortical neurons (14 DIV) and quantification normalized to Vinculin and β-III Tubulin. (**B**,**C**) Representative phase and fluorescence image (**B**) and quantified soma area (**C**) of shControl and shPum2 transfected mature cortical neurons (14 DIV, >38 cells were measured for each condition, *n* > 4 cultures). (**D**–**F**) Representative microscopy images (**D**) and Sholl analysis (**E**,**F**) of shControl and shPum2 transfected cortical neurons. (**G**,**H**) Representative immunoblot against phospho-mTOR (Ser2448) (**D**) and quantification from control and shPum2 transduced neuronal lysates (**E**). β-III tubulin was used as loading control. Arrow heads indicate the respective proteins. *p*-values were calculated using Mann–Whitney test (**C**) or unpaired Student’s *t*-test (**A**,**F**). * *p* < 0.05, ** *p* < 0.01, *** *p* < 0.001, Scale bar: 10 µm.

To test this hypothesis, we exploited a well-characterized shRNA construct [11] to specifically downregulate Pum2 but not its paralog Pum1 [18]. Therefore, cortical neurons were transduced with lentiviruses expressing either shControl or shPum2 constructs. Upon 4 days of incubation, Pum2 levels were analyzed by Western blotting. In agreement with previous findings [11,18], we observed a strong downregulation of Pum2 (Figure 2A). To test whether Pum2 affects neuronal growth, we transfected mature cortical neurons (10 DIV) with constructs containing a separate GFP open reading frame expressing either shPum2 or a scrambled control shRNA. Upon 4 days of downregulation, we measured soma area as an approximation for cellular growth. Interestingly, loss of Pum2 led to reduced soma area (Figure 2B,C). To complement these findings, we analyzed dendritic branching exploiting Sholl analysis as previously described [11]. Interestingly, and in line with the soma growth deficit, we observed less branching of Pum2 depleted cortical neurons (Figure 2D–F). Importantly, this effect was independent of mechanistic target of rapamycin (mTOR) activity, as its phosphorylation at Ser2448 remained unchanged upon Pum2 KD (Figure 2D,E) suggesting an effect downstream of mTOR. Interestingly, we observed a similar growth defect in yeast cells lacking the Pum2 homolog Puf3 indicating an evolutionary conserved role (Figure S1D [25]). Therefore, our results demonstrate that Pum2 is needed for growth control in neurons downstream of the mTOR signaling pathway.

### 2.3. Pum2 Activates Translation of eIF4E

**Figure 3:**
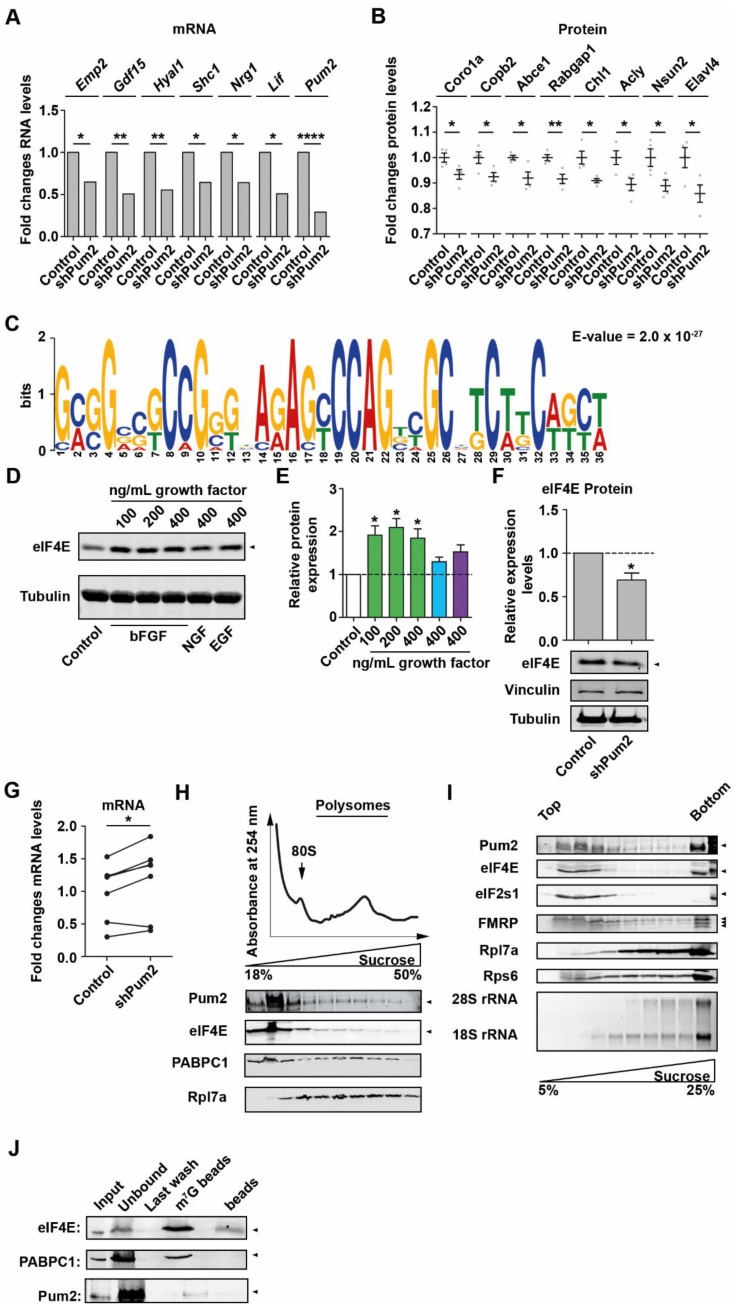
Pum2 promotes *eIF4E* translation. (**A**,**B**) RNA (**A**) and protein (**B**) fold changes of growth regulators upon knock-down of Pum2 (data extracted from the work in [18]). (**C**) Consensus sequence and E-value of the 5′-UTR of Pum2 regulated mRNAs identified by MEME analysis. (**D**,**E**) Representative immunoblot (**D**) against eIF4E and quantifications I upon incubation of mature (14 DIV) cortical neurons with bFGF, NGF, and EGF, respectively, for two days. β-III Tubulin was used as loading control. (**F**) Representative immunoblot against eIF4E from shControl and shPum2 transduced cortical neurons (14 DIV) and quantification normalized to Vinculin and β-III Tubulin. (**G**) Relative *eIF4E* mRNA levels in shControl and shPum2 transduced neurons. *Peptidyl-prolyl cis-trans isomerase A* (*PPIA*) was used as reference gene. Line connects control and Pum2 depleted conditions from identical cultures. (**H**) Representative polysome profile (18–50%) of post-nuclear brain lysate and representative immunoblots for Pum2 (eIF4E and PABPC1 served as marker for the translation initiation machinery, Rpl7a for ribosomes). (**I**) Representative immunoblot for Pum2 upon centrifugation through high-resolution sucrose gradients (5–25%). eIF2s1 and eIF4E served as markers for the translation initiation machinery, Rpl7a and Rps6 for large and small ribosomal subunits, respectively, FMRP is a Pum2 protein interactor. (**J**) Representative immunoblot against eIF4E, PAPBC1, and Pum2 upon incubation with m^7^G beads. Arrow heads indicate the respective proteins. *p*-values were calculated using Wald test (**A**), unpaired Student’s *t*-test (**B**), and one-sample *t*-test (**E**,**F**). * *p* < 0.05, ** *p* < 0.01, **** *p* < 0.0001, 3–4 biological replicates.

To unravel the molecular mechanism of how Pum2 might regulate neuronal growth, we exploited a recently generated transcriptome and proteome dataset of Pum2-depleted cortical neurons of the same age [18]. We selected those genes with a direct link in controlling growth of neurons or tumor cells. Interestingly, we found six genes with downregulated mRNA and eight growth regulators with downregulated protein levels (Figure 3A,B). Among those are essential regulators of growth and development such as the SHC-transforming protein 1 (Shc1) [26], Neuregulin 1 (Nrg1) [27,28,29], Coronin 1a (Coro1a) [30], and the Rab GTPase-activating protein 1 (Rabgap1) [31]. As next step, we asked whether there is (a) global regulator(s) that (i) has the potential to control expression of a large number of genes, (ii) is involved in growth regulation, and (iii) regulated and bound by Pum2. To identify a potential regulator, we first analyzed sequence motifs in regulated mRNAs. Therefore, we focused on proteins that were significantly downregulated in Pum2 depleted neurons [18]. We analyzed the 5′-UTR of their corresponding mRNAs as it contains important regulatory motifs that control translation initiation [32]. Using MEME [33], we found that the 5′-leader of these transcripts exhibit an enrichment of guanines and cytosines (Figure 3C). Interestingly, mRNA targets of eIF4E show an enrichment of cytosines in the 5′-UTR [34]. This finding led us to speculate that eIF4E might regulate growth in neurons in a Pum2-dependent manner. To test this hypothesis, we first tested whether eIF4E expression is regulated by bFGF. Interestingly, and similar to Pum2, we found eIF4E to be upregulated upon bFGF but not upon NGF or EGF treatment (Figure 3D,E), indicating that eIF4E is needed for growth stimulation. In line with this notion are published findings showing that eIF4E critically regulates cellular growth [17,35]. To unravel the impact of Pum2 on eIF4E expression, we analyzed eIF4E levels in Pum2-depleted lysates. We found that depletion of Pum2 led to a significant downregulation of eIF4E protein, while *eIF4E* mRNA was upregulated (Figure 3F,G). This indicates a drop in translation activity of the *eIF4E* transcript, which is in line with our previous finding that Pum2 activates global translation [18].

To investigate whether Pum2 regulates eIF4E protein or its corresponding mRNA, we first biochemically characterized Pum2 granules using differential centrifugation similar to what has been done for Stau2 [36]. Interestingly, Pum2 complexes were preferentially found in the pellet (P) fraction compared to supernatant (S) upon 100,000× *g* centrifugation (Figure S2A). Strikingly, RNase1 treatment prior to centrifugation released Pum2 into the supernatant, whereas EDTA or DTT treatment did not, indicating that its association was RNA mediated (Figure S2A). This is not a global feature of all RBPs, as two other RBPs found in RNA granules, Barentsz (Btz) [37] and the nuclear protein NeuN (Rbfox3) [38], were insensitive to RNase1 treatment. To further investigate a possible association of Pum2 with cellular components, we employed sucrose cushion centrifugation. Again, we found that Pum2 was enriched in the pellet fraction together with ribosomal proteins (Figure S2B). Most likely, Pum2 interacts with cytosolic ribosomes rather than those associated with the endoplasmic reticulum (ER), as Sec61A, a marker for ER, migrates differently during centrifugation. To further confirm the interaction of Pum2 with the translation machinery, we performed polysome profiling using post-nuclear brain lysates. The majority of Pum2 did not co-migrate with polysomes but was enriched in translationally dormant fractions. Another fraction of Pum2, however, co-migrated with polysomes (Figure 3H). Further, Pum2 accumulated in subpolysomal fractions positive for translation initiation factors such as eIF4E. To better distinguish between monosomes and ribonucleoprotein particle (RNP) fractions, we exploited high-resolution sucrose gradient centrifugation to better resolve Pum2 containing particles [39]. Interestingly, Pum2 was found to be enriched in fractions free of ribosomes but containing translation initiation factors (eukaryotic initiation factor 2 subunit 1 (eIF2s1), eIF4E) as well as FMRP, a known Pum2 protein interactor [6] (Figure 3I). A significant fraction of Pum2, however, was found in the pellet fraction together with ribosomal proteins. As control, we performed mRNA cap (m^7^G) pulldowns to enrich for the translation initiation complex revealing that Pum2 is not an integral component of the initiation complex (Figure 3J). Our data indicate that Pum2 is not part of the translation initiation machinery. Thus, our results suggest that Pum2 regulates eIF4E at the mRNA rather than at the protein level. 

### 2.4. Pum2 Binds eIF4E mRNA

**Figure 4:**
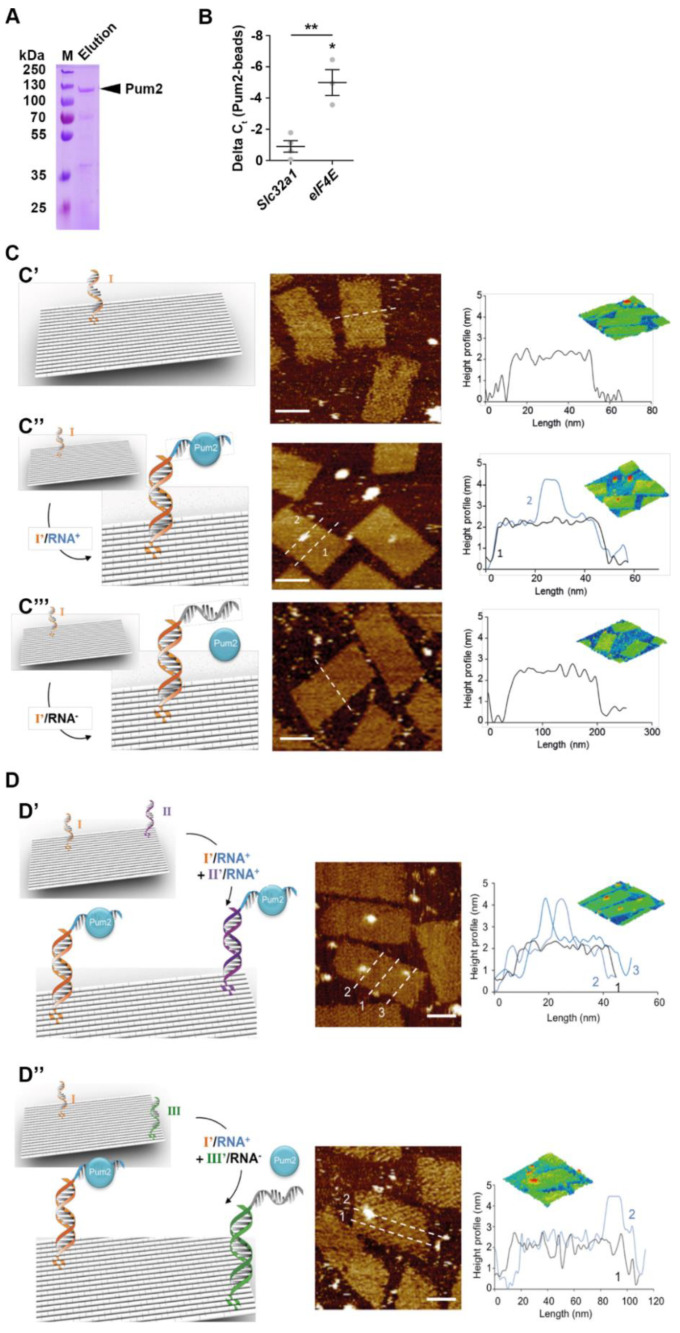
Atomic force microscopy reveals Pum2 binding to *eIF4E* mRNA. (**A**) Coomassie-stained SDS-gel upon Nickel-NTA purification of His_6_-tagged full length Pum2. (**B**) Relative enrichment of *eIF4E* and *Slc32a1* mRNA in His_6_-tagged full length Pum2 pulldown. (**C**,**D**) Scheme for AFM experimental setup (**left**), representative AFM images (**middle**), and height profiles (**right**) for Pum2 particles bound to immobilized *eIF4E* 3′-UTR RNA probes. See text for explanations. *P*-values were calculated using one-sample *t*-test within samples and unpaired Students *t*-test between samples. * *p* < 0.05, ** *p* < 0.01, 4 biological replicates.

Next, we asked whether *eIF4E* is a direct mRNA target of Pum2. Using electrophoretic mobility assays with purified Pum2 protein and *eIF4E* RNA, it has been shown that it binds the 3′-UTR of *eIF4E* [11,22]. However, RBPs need to select their targets among thousands of different transcripts within cells. We therefore used recombinantly expressed and purified Pum2 protein (Figure 4A) together with total RNA isolated from rat brain to perform pulldown experiments followed by qRT-PCR analysis. Interestingly, and in line with recent studies, we observed an enrichment of *eIF4E* mRNA in Pum2 pulldown but not in control eluates. As control mRNA, we chose the *solute carrier family 32 member 1* (*Slc32a1*) coding for vesicular GABA transporter (Vgat), a protein that is affected upon Pum2 downregulation [18]. Interestingly, *Slc32a1* was not enriched (Figure 4B). Next, we asked whether *eIF4E* mRNA is sufficient to recruit Pum2 protein. Therefore, we exploited atomic force microscopy (AFM). A fragment of *eIF4E* 3′-UTR containing the Pum2 binding consensus sequence (UGUANAUA) [40] was immobilized using the DNA origami technology [41] and incubated with purified Pum2 (Figure 4C,D). Strikingly, we observed RNA granule-like complexes on the origami (Figure 4C’’) that were absent on the surface when the *eIF4E* 3′-UTR was omitted (Figure 4C’). As negative control, we inverted the Pum2 binding motif leading to diminished Pum2 binding (Figure 4C’’’). To test for specific binding, we immobilized RNA probes at different positions on the DNA origami (Figure 4D). Interestingly, Pum2 followed this change in localization, ruling out that the effect is mediated due to unspecific Pum2 binding (Figure 4D’,D’’). These results strongly suggest that *eIF4E* is selectively bound by Pum2 to assemble into RNA granules.

## 3. Discussion

Cells control their physiological homeostasis through different global regulators such as transcription and translation factors, kinases, phosphatases, and—last but not least—RBPs. The complex interaction between these molecules allow cells to proceed through development, to grow and to respond to external stimuli [42,43]. Here, we show that Pum2 regulates growth and dendritic branching of mature neurons downstream of the bFGF/mTOR signaling pathway. At the molecular level, it activates translation of eIF4E to enhance global translation [18]. Notably, the effect of Pum2 on eIF4E expression might depend on the cell type and maturity of neurons. In *Drosophila* and rat immature neurons, Pum2 represses eIF4E expression [11,22]. In line with this notion is the finding that Pum2 deficiency decreases dendritic branching in cortical neurons. In hippocampal neurons, however, deletion leads to increased branching [11]. Future studies are clearly needed to unravel the cell type-specific effect of Pum2 and its molecular consequences.

The coordinated regulation of protein synthesis has important consequences for the cell including growth [42]. We previously described that Pum2 activates global translation in cortical neurons [18]. In our study, we observed a reduction in cell body area of mature neurons. Interestingly, this effect was not caused by a dysregulated mTOR pathway, which represents a crucial regulatory hub for growth [43]. Thus, it is plausible that reduced translational activity account for the observed growth deficit. In addition, we found important growth regulators downregulated in Pum2-deficient neurons. In this context, Coronin 1a is of particular interest as it interacts with and, in turn, organizes the actin cytoskeleton network [44]. Dysregulation of the actin cytoskeleton can be causative for growth defects [45]. Moreover, RBPs such as Stau2 and FMRP control actin cytoskeleton organization [46,47], pointing towards a common regulatory hub mediated by posttranscriptional regulators. Interestingly, we found that transcripts that are translationally downregulated upon Pum2 knockdown [18] share binding motifs in their 5′-UTRs that might be regulated by the eIF4E [34]. It has been shown that eIF4E is essential for growth [17]. Moreover, our data suggest that Pum2 binds *eIF4E* mRNA and enhances its translation. Notably, while eIF4E protein is downregulated, its corresponding transcript increases. This effect reflects a cellular compensation strategy to counteract lower levels of essential translation initiation factors. Additionally, and not mutually exclusive, eIF4E protein levels might be downregulated as response to translational buffering [48]. In this context, the translational machinery buffers transcript fluctuations to maintain protein levels. In any case, it is plausible that Pum2 regulates both translation and growth through eIF4E. Importantly, our data are consistent with previously published data on Pumilio proteins. Here, it has been shown that Pum1 and Pum2 regulate body size and neurogenesis [6,12]. Therefore, our data provide a possible mechanistic explanation for these observations.

In addition to nerve cells, Pum2 is highly expressed in different cancer cells. Interestingly, in glioblastoma and breast cancer cells, it is needed for proliferation and stemness maintenance, respectively [14,15]. Moreover, *eIF4E* is needed for efficient oncogenic transformation of cells [34]. Thus, Pum2 might be important for oncogenic transformation as well. Interestingly, cancer cells derived from breast cancer synaptically integrate to promote metastasis formation in the brain [49]. Importantly, Pum2 regulate neuronal excitability [50] as well as neuronal inhibition [18], indicating a role in balancing synaptic activity. Due to its high expression in cancer cells, it is tempting to speculate that Pum2 is also needed for integration of cancer cells into neuronal circuits. Future experiments are clearly needed to unravel the role of Pum2 in regulating cancer metastasis formation.

## 4. Materials and Methods

### 4.1. Animals

Pum2 gene trap (Pum2^GT^) and WT mice (background: C57Bl6/J (Charles River, Cologne, Germany) and C57Bl6/JRccHsd (Envigo, Bresso, Italy)) were used throughout. All mice were kept under specified pathogen-free conditions and housed in groups of 3 animals in individually ventilated cages (IVC) and a 12 h/12 h light/dark cycle. Mice had free access to water (acidified and desalinated) and standard rodent chow (Altromin, 1310 M). 

### 4.2. Differential Centrifugation and Sucrose Cushion Centrifugation

One brain hemisphere of postnatal day 21 (P21) mouse (Bl6/J) was homogenized in homogenization buffer (HB; 150 mM KCl, 50 mM Hepes pH 7.4, 1× complete protease inhibitor (Roche), 5 µL Ribolock (ThermoFisher, Darmstadt, Germany) per 10 mL HB) on ice using a hand-driven glass douncer. Homogenate was spun at 16,000× *g* for 10 min at 4 °C (S16, P16). Supernatant S16 was spun at 100,000× *g* for 20 min at 4 °C (S100, P100). When indicated, samples were treated with RNase1 prior to centrifugation. P100 pellets were volume-even resuspended in RIPA buffer (150 mM NaCl, 50 mM Tris-HCl pH 8, 0.5% (*w*/*v*) sodium deoxycholate, 1 vol% NP-40, 0.1% (*w*/*v*) SDS, 1× complete protease inhibitor (Roche, Germany)) at 37 °C. All fractions (S16, P16, S100, P100) were methanol/chloroform extracted as described [51]. Pum2 and Btz, respectively, were detected using SDS PAGE and Western blotting.

For sucrose cushion centrifugation, one P21 mouse brain was homogenized in polysome lysis buffer (150 mM NaCl, 5 mM MgCl_2_, 10 mM Tris-HCl pH 7.4, 1 vol% NP-40, 1% (*w*/*v*) sodium deoxycholate supplemented with 100 µg/mL cycloheximide (CHX) and 2 mM dithiothreitol, DTT) at 4 °C as described above. Lysate was spun at 13,000× *g* for 5 min at 4 °C. Postnuclear lysate was loaded on 2 mL 20% sucrose cushion (20% (*w*/*v*) sucrose, 100 mM KCl, 5 mM MgCl_2_, 20 mM Hepes pH 7.4) and centrifuged for 2 h at 100,000 rpm (SW41Ti rotor, Beckman Coulter, Germany) at 4 °C. Pellets were resuspended in SDS Laemmli buffer, separated by SDS PAGE, and transferred to nitrocellulose by Western blotting.

### 4.3. m^7^GTP Pulldown

One P21 mouse brain hemisphere was lysed in 1× brain extraction buffer (BEB) [52] using a hand-driven douncer. Lysate was spun at 20,000× *g* (S20) for 15 min at 4 °C. S20 was precleared with protein G sepharose beads. For pulldown, beads (m^7^GTP beads (Jena Bioscience, Jena, Germany) or agarose beads as control) were washed 4 times in PBS and once in BEB. Then, beads were incubated in blocking solution (1.5 mg/mL BSA in 1× BEB) for 1 h at 4 °C. S20 supplemented with 100 µM GTP as competitor was incubated with beads for 1 h at 4 °C. Unbound proteins were removed by washing beads three times with BEB. Proteins were eluted with 0.2 M glycine supplemented with 400 mM arginine and then TCA precipitated.

### 4.4. Recombinant Pum2 Expression and Purification

His-tagged full-length murine Pum2 (Uniprot ID: Q80U58-2) was expressed in *E.coli* (Rosetta pLysS) cells (10 L culture). Expression was induced with 0.2 mM isopropyl-β-D-thiogalactopyranosid (IPTG) at OD_600 nm_~0.5 (exponential phase) for 2 h at 37 °C. Cells were harvested and lysed in lysis buffer (1× PBS supplemented with 880 mM NaCl, 400 mM arginine, 2 mM DTT, 1× complete protease inhibitor (Roche), 500 µM phenylmethanesulfonyl fluoride) using ultrasound. Lysate was spun at 15,000 rpm for 1 h at 4 °C in a JA 25.5 rotor (Beckman Coulter). Pellet was resuspended in lysis buffer, sonicated, and then centrifuged again. Supernatants were pooled and adjusted to pH 7.6. Precleared lysates were incubated with Nickel NTA beads (Qiagen), then washed with two column volumes in lysis buffer. Proteins were eluted with a continuous elution buffer gradient (lysis buffer supplemented with 0 to 300 mM Imidazole). Eluate was concentrated (Vivaspin 20, 10 kDa). Sample buffer was changed to Hepes buffer (20 mM Hepes pH 7.5, 150 mM NaCl, 2 mM DTT, 50 mM arginine). Then, elution was loaded on a Superose6 column for gel filtration. Proteins were eluted in Hepes buffer (20 mM Hepes pH 7.5, 150 mM NaCl, 2 mM DTT, 50 mM arginine). Proteins were concentrated and buffer changed to PBS. Proteins were frozen at −80 °C for long-term storage.

### 4.5. RNA Pulldown

For RNA pull-down, Nickle-NTA beads were first blocked in blocking reagent (Roche) supplemented with 1 µg/µL yeast tRNA for 1 h at 4 °C. Approximately 100 µg purified Pum2 protein was incubated with 4 µg rat brain RNA in PBS supplemented with RNase inhibitor for 1 h at 4 °C. Pum2-RNA complexes were then incubated with blocked Nickel-NTA beads for 1 h at 4 °C. Empty beads were used as control. Beads were washed 4 times in PBS. DNA digestion and cDNA synthesis [10] were performed on beads.

### 4.6. Quantitative Real-Time PCR (qRT-PCR)

RNA was isolated from DIV14 cultured cortical neurons using TRIzol according to the manufacturer’s manual. cDNA synthesis and qRT-PCR were performed as described [10]. Primers were tested and optimized for an efficiency of 2 ± 0.05. For relative quantification, the ΔΔCq method was used with *PPIA* as reference gene [53].

### 4.7. Polysome Profiling

Polysome profiling was performed as previously described [18]. In brief, one brain hemisphere of a juvenile mouse was homogenized in polysome lysis buffer as reported above. Lysates were precleared at 13,000× *g* at 4 °C for 5 min and subsequently loaded onto sucrose gradients (18% (*w*/*v*) to 50% (*w*/*v*) sucrose in 100 mM KCl, 5 mM MgCl_2_, 20 mM Hepes pH 7.4). Upon centrifugation at 35,000 rpm (SW55Ti rotor, Beckman Coulter) at 4 °C for 1.5 h, gradients were fractionated (Piston Fractionator, Biocomp, Munich, Germany) with continuous RNA fate detection at 254 nm. Proteins were extracted using methanol/chloroform extraction [51].

### 4.8. Western Blotting and Antibodies

Lysates were separated by SDS-PAGE. Proteins were transferred to nitrocellulose (pore size 0.2 µm). Membranes were blocked in blocking solution (2% (*w*/*v*) BSA, 0.1 vol% Tween 20, 0.1% (*w*/*v*) sodium azide in 1× TBS pH 7.5) for at least 1 h. Primary antibodies were diluted in blocking buffer and incubated overnight at 4 °C. The following antibodies were used in this study: polyclonal antibodies: rabbit anti-Pum2 (Abcam) 1:10,000, rabbit anti-Btz (self-made, [52]) 1:500, rabbit anti-Rpl7a (Abcam) 1:1000, rabbit anti-Rps6 1:1000, rabbit anti-phospho-Rps6 1:1000, rabbit anti-PABP1 (all Cell Signaling) 1:1000, goat anti-Vinculin (Santa Cruz) 1:200; monoclonal antibodies: mouse anti-eIF4E (BD) 1:1000, mouse anti-eIF2s1 (Cell Signaling) 1:1000, mouse anti-FMRP (gift from Utz Fischer, Würzburg) 1:1000, mouse anti-β-III-Tubulin (Sigma Aldrich) 1:10,000, and rabbit anti-phospho-mTOR (Cell Signaling) 1:1000).

Membranes were washed in PBS supplemented with 0.2 vol% Tween 20. Primary antibodies were detected using infrared dye labeled secondary anti-rabbit, anti-goat, or anti-mouse antibodies (all 1:10,000, Li-COR Biosciences). Membranes were scanned using the Li-Cor Odysey IR scanner.

### 4.9. DNA Origami

The analysis of protein binding to the proposed RNA consensus sequence was carried out using the DNA origami method [41]. The DNA origami structures were assembled using a 1:10 molar ratio between the M13mp18 viral DNA (10 nM) and each of the staple strands, in 1× TEMg buffer. All staples used for the self-assembly are reported in Table S1. Thermal annealing was performed by decreasing the temperature from 70 °C to 20 °C at −1 °C min^−1^ on a Thermocycler Mastercycler nexus gradient (Eppendorf). In a second step, the DNA origami hosts were decorated on their surface with one of four distinct DNA-RNA mixed sequences (full nomenclature of the systems used in this work is given in Supplementary Figure S1). Functionalization of the DNA origami surface was performed through hybridization of the DNA segment of the DNA–RNA mixed strand to a complementary DNA handle protruding out of the origami surface and pointing towards the solution. After thermal annealing at mild conditions (from 40 °C to 20 °C at −1 °C min^−1^), the structures were incubated with a 3-fold excess of Pum2 for 1 h at room temperature (RT). To remove the excess of staples and unreacted RNA-protein conjugates from the modified origami solution, PEG purification was used as previously described [54]. Finally, the purified DNA origami–protein complexes were suspended in BEB-buffer and analyzed by atomic force microscopy (AFM). 

All oligonucleotides were purchased from Sigma-Aldrich as desalted products and delivered lyophilized in 96-well plates. All solutions were prepared using Milli-Q water as the solvent (Milli-Q^®^ Integral Water Purification System) and further filtered on 0.22 μm membrane filters (cellulose acetate, sterile, cat. # 28145-477) supplied by VWR. Single-stranded M13mp18 DNA, propagated in *E.coli* XL1-Blue (Agilent technologies; cat. # 200249) was produced from phage DNA (Affymetrix; cat. # 71706) as previously reported [55]. Buffers used were 1× TEMg (20 mM Tris base, 2 mM EDTA, 12.5 mM MgCl_2_, pH 7.6), 1× TAE (40 mM Tris base 20 mM acetic acid, 2 mM EDTA, 12.5 mM magnesium acetate, pH 8), 1× PEG-Buffer (5 mM Tris base, 1 mM EDTA, 505 mM NaCl, 15% (*w*/*v*) PEG 8.000) and 1× BEB (25 mM HEPES, 150 mM KCl, 8% glycerol, 0.05% NP-40, pH 7.3).

### 4.10. Atomic Force Microscopy

The DNA origami sample (5 μL of a 1:10 solution in TAE buffer) was deposited on a freshly cleaved mica surface (Plano GmbH) and adsorbed for 3 min at RT. The sample was then mounted on top of the microscope scanner and imaged in fluid upon addition of 15 μL of TAE buffer. AFM imaging was performed using a MultiModeTM 8 microscope (Bruker) equipped with a Nanoscope V controller, using the ScanAsyst^®^ PeakForce TappingTM operational mode. Silicon nitride probes with 0.7 N/m nominal spring constant and sharpened pyramidal tips (ScanAsyst Fluid+, Bruker) were used for scanning. Several AFM images were acquired from different locations of the mica surface to ensure reproducibility of the results. All images were analyzed by using the NanoScope 6.14 and Gwyddion 2.45 software. Binding yields are reported in Figure S1.

### 4.11. Neuronal Cell Culture and Transfection

Neuronal cell culture from rat was performed as previously described [46] with slight modifications for cortical neurons. For transient transfection of shPum2 and control plasmids [11], DNA calcium phosphate co-precipitation was performed as previously described [56]. Lentivirus transduction was performed as previously described [18,57].

### 4.12. Growth Factor Incubation of Cortical Neurons

Mature cortical neurons (10/11 DIV) were incubated with indicated concentrations of bFGF, EGF and NGF (Peprotech) for two days. Upon incubation, cells were washed three times in warm HBSS and lysed in hot SDS sample buffer. 

### 4.13. Yeast Strains and Growth Conditions

*Saccharomyces cerevisiae* strain YPH499 was used as wild type strain. The deletion of the open reading frame encoding for Puf3 was performed according to standard procedures with slight modifications [58]. Kluyveromyces lactis LEU2 was inserted into the open reading frame coding for *Puf3*. Cells were grown as indicated on YPD (1% yeast extract, 2% peptone, 2% dextrose) or YPG (1% yeast extract, 2% peptone, 3% glycerol) at 30 °C [59]. 

### 4.14. Growth Analysis of Yeast Strains

Wild type and Puf3 deletion mutant cells were grown in liquid medium and kept in logarithmic growth phase. Cells were harvested by centrifugation (10 min at 3000× *g* and RT), washed once with water, and diluted in water to an OD_600_ of 0.3. Starting from this cell suspension, serial dilutions were performed (1:10; 1:100; 1:1000). Three microliters of each dilution was spotted on agar plates containing either YPD or YPG medium and were incubated at 30 °C.

### 4.15. Immunostaining and Image Analysis

Upon fixation with 4% PFA, cells were washed with HBSS and mounted in Fluoromount (Sigma Aldrich). Fluorescence microscopy was performed using the Observer Z1 microscope (Zeiss) with a 63× planApo oil immersion objective (1.40 NA). For analysis of dendritic branching, GFP images were used. Sholl analysis was performed using ImageJ.

### 4.16. RNA Expression Analysis

For RNA expression analysis of RBPs, we extracted RNA-seq data from the Expression Atlas Homepage (https://www.ebi.ac.uk/gxa/experiments/E-MTAB-2770/Results, accessed 01/2021). Expression data was used from the following cell lines: 42-MG-BA, 8-MG-BA, A172, AM-38, CAS-1, DBTRG-05MG, DK-MG, GAMG, GB-1, GMS-10, GOS-3, KALS-1, KNS-42, KNS-60, KNS-81, KS-1, LN-18, LN-229, M059K, SF-295, SF126, SNB75, SNU-1105, SNU-201, SNU-466, SNU-489, SNU-626, T98G, U-87 MG, YH-13, YKG1 for glioblastoma, CHP-126, CHP-212, IMR-32, KELLY, KP-N-RT-BM-1, KP-N-SI9s, KP-N-YN, MHH-NB-11, NB-1, SIMA, SK-N-AS, SK-N-BE(2), SK-N-DZ, SK-N-FI for neuroblastoma and CAL-51, DU4475, EFM-192A, HDQ-P1, MC-1-8, Hs 281.T, Hs 343.T, Hs 606.T, Hs 739.T, Hs 742.T, JIMT-1, MDA-MB-157 for breast cancer.

### 4.17. Statistics

For data analysis and statistics, the prism software (version 5 GraphPad, San Diego, CA, USA) was used. Data were tested for normal distribution using Kolmogorov–Smirnov or Shapiro–Wilk normality tests. To calculate *p*-values, paired or unpaired Student’s *t*-test and Mann–Whitney test were used, respectively. To test for significance within samples, one-sample *t*-test was used. To compare multiple groups with each other, Kruskal–Wallis test with Dunn’s Multiple Comparison Test or Tukey’s Multiple Comparison Test were exploited. To analyze transcriptome data from cortical neurons [18], Wald test was used. *p* < 0.05 was considered as statistically significant.

## 5. Conclusions

In conclusion, our study suggests that Pum2 acts as a neuronal growth factor to allow sufficient expression of important transcripts, e.g., eIF4E. This is an essential requirement for neuronal functioning. Future studies are clearly needed to unravel the molecular pathways underlying growth, development, and synaptic plasticity that are all controlled by Pum2. Based on our findings, and due to its broad impact on cellular growth of different cell types, it is therefore likely to be a potential anticancer target for gene therapies.

## Supplementary Materials

The following files are available online at https://www.mdpi.com/article/10.3390/ijms22168998/s1.

## Abbreviations

AFM	Atomic force microscopy
BDNF	Brain derived neurotrophic factor Semaphorin 3A (Sema3A)
Btz	Barentsz
bFGF	basic fibroblast growth factor
Coro1a	Coronin 1a
4E-T	Eukaryotic initiation factor 4E transporter
eIF4E	Eukaryotic initiation factor 4E
eIF2s1	Eukaryotic initiation factor 2 subunit 1
EGF	Epidermal growth factor
FMRP	Fragile-X mental retardation protein
MEME	Multiple EM for Motif Elicitation
mTOR	Mechanistic target of rapamycin
Nrg1	Neuregulin 1
NGF	Neuronal growth factor
Pum1	Pumilio1
Pum2	Pumilio2
Rabgap1	Rab GTPase-activating protein 1
Rbfox1	RNA-binding protein fox-1 homolog 1
Sema3A	Semaphorin 3A
Shc1	SHC-transforming protein 1
Slc32a1	Solute carrier family 32 member 1
Stau1	Staufen1
Stau2	Staufen2
