# Supplementary material for: Pumilio2 Promotes Growth of Mature Neurons
Source: Int J Mol Sci. 2021 Aug 20;22(16):8998. doi: 10.3390/ijms22168998 (PMC8396670; doi:10.3390/ijms22168998)
Supplement: Supplementary file 1 [file ijms-22-08998-s001.zip › ijms-1318388-supplementary.pdf]

Supplementary Figure 1: Pum2 enhances growth in yeast

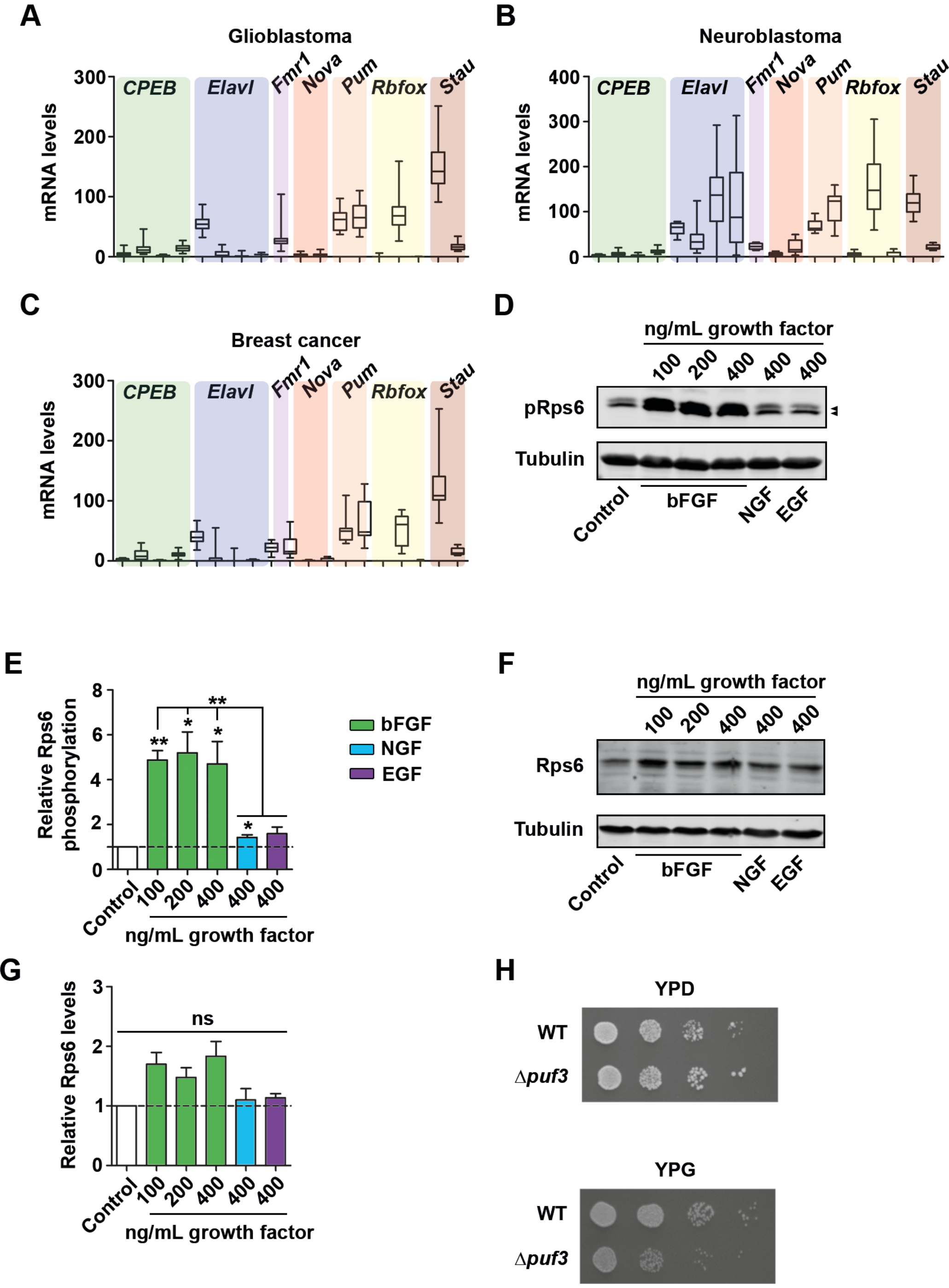

Figure S1: Pum2 enhances growth in yeast. (**A**-**C**) Transcript levels (TPM) of essential RBP families in glioblastoma (**A**), neuroblastoma (**B**) and breast cancer cells (**C**). (**D**-**G**) Representative immunoblot against pRps6 (**D**) and Rps6 (**F**) with respective quantifications (**E**,**G**) upon incubation of mature (14 DIV) cortical neurons with bFGF, NGF and EGF, respectively, for two days. β-III-Tubulin was used as loading control. Arrow heads indicate the respective proteins. (**H**) Yeast wild type and PUF3 depleted cells were grown in liquid medium and kept in logarithmic phase. The cultures were diluted to an OD600 of 0.3 and serial dilutions were performed (1:10, 1:100, 1:1,000). 3 µL of each dilution were spotted on culture plates containing rich YP medium supplemented with dextrose (YPD) to allow yeast to grow under fermentative conditions or glycerol (YPG) to force yeast to respire. P-values were calculated using one-sample t-test within samples and Tukey's Multiple Comparison Test between samples. $^{*}p < 0.05$, $^{**}p < 0.01$, 3-4 biological replicates.

Supplementary Figure 2: Pum2 complexes are RNase-sensitive

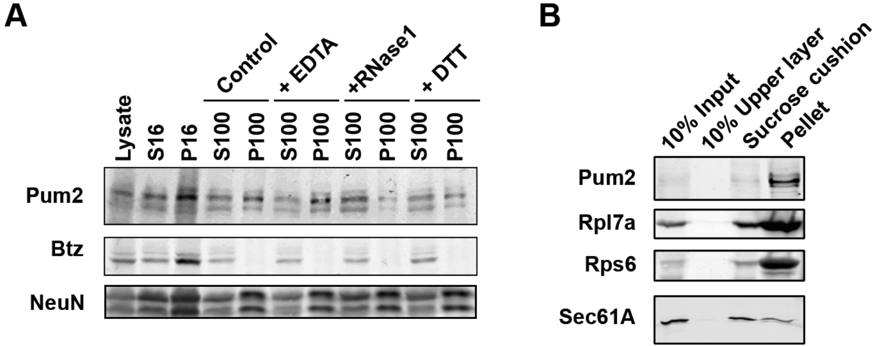

Figure S2: Pum2 complexes are RNase-sensitive. (**A**) Representative Western Blot showing Pum2, Btz and neuronal nuclei antigen (NeuN) in pellet and supernatant upon differential centrifugation of brain lysates. (**B**) Representative Western blot of Pum2 upon sucrose cushion centrifugation (Rpl7a, Rps6 served as markers for ribosomes, Sec61A for ER).

Supplementary Figure 3: Sequences used for AFM

A

Oligonucleotide	Sequence 5‘-3‘
I’/RNA+	AGUGUACAAAAC***UGUAAAUA***AAAUGUGUACAG**CGACGACGACGACGA**
I’/RNA-	AGUGUACAAAAC***AUAAAUGU***AAAUGUGUACAG**CGACGACGACGACGA**
II’/RNA+	AGUGUACAAAAC***UGUAAAUA***AAAUGUGUACAG**TTGTTGTTGTTGTTG**
III’/RNA-	AGUGUACAAAAC***AUAAAUGU***AAAUGUGUACAG**TACTACTACTACTAC**
I	TTGAGGATATTGTTTGGATTATACATAACGGA**TCGTCGTCGTCGTCG**
II	CCGCTTTCTAAAGTGTAAAGCCTGCCATTCGC**CAACAACAACAACAA**
III	TGAGGAAGCGAAAGACAGCATCGGTTTTGCTA**GTAGTAGTAGTAGTA**

B

DNA handle	DNA-RNA antihandle	Yield (%)
I	-	0±0
I	I’/RNA^{+}	14±6
I	I’/RNA^{-}	1.8±1
I + II	I’/RNA^{+}<br>II’/RNA^{+}	10±3
I + III	I’/RNA^{+}<br>III’/RNA^{-}	13±3

Figure S3: Sequences used for AFM. (**A**) List of DNA and DNA/RNA mixed oligonucleotides used in this work. The RNA sequence is written in bold and italics (a “+” or “-“ subscript indicates, respectively, a sense or antisense RNA sequence). The protein is supposed to bind exclusively to the RNA+ sequence. The DNA handles (protruding out of the DNA origami surface) and antihandles (covalently attached to the RNA sequence) are indicated, respectively, as I/I’, II/II’ and III/III’. The complementary DNA segments are highlighted in bold. (**B**) AFM yields of purified complexes indicate successful binding of the Pum2 protein.
